# Characterization of three different sensory fibers by use of neonatal capsaicin treatment, spinal antagonism and a novel electrical stimulation-induced paw flexion test

**DOI:** 10.1186/1744-8069-2-16

**Published:** 2006-05-08

**Authors:** Misaki Matsumoto, Makoto Inoue, Andreas Hald, Asuka Yamaguchi, Hiroshi Ueda

**Affiliations:** 1 Division of Molecular Pharmacology and Neuroscience, Nagasaki University Graduate School of Biomedical Sciences, Nagasaki 852-8521, Japan

## Abstract

In the present study, we first report an *in vivo *characterization of flexor responses induced by three distinct sine-wave stimuli in the electrical stimulation-induced paw flexion (EPF) test in mice. The fixed sine-wave electric stimulations of 5 Hz (C-fiber), 250 Hz (Aδ-fiber) and 2000 Hz (Aβ-fiber) to the hind paw of mice induced a paw-flexion response and vocalization. The average threshold for paw flexor responses by sine-wave stimulations was much lower than that for vocalization. Neonatally (P3) pretreatment with capsaicin to degenerate polymodal substance P-ergic C-fiber neurons increased the threshold to 5 Hz (C-fiber) stimuli, but not to 250 Hz (Aδ-fiber) and 2000 Hz (Aβ-fiber). The flexor responses to 5 Hz stimuli were significantly blocked by intrathecal (i.t.) pretreatment with both CP-99994 and MK-801, an NK1 and NMDA receptor antagonist, respectively, but not by CNQX, an AMPA/kainate receptor antagonist. On the other hand, the flexor responses induced by 250 Hz stimuli were blocked by MK-801 (i.t.) but not by CP-99994 or CNQX. In contrast, flexor responses induced by 2000 Hz stimuli were only blocked by CNQX treatment. These data suggest that we have identified three pharmacologically different categories of responses mediated through different primary afferent fibers. Furthermore, we also carried out characterization of the *in vivo *functional sensitivity of each of the sensory fiber types in nerve-injured mice using the EPF test, and found that the threshold to both 250 Hz and 2000 Hz stimulations were markedly decreased, whereas the threshold to 5 Hz stimulations was significantly increased. Thus we found opposing effects on specific sensory fiber-mediated responses as a result of nerve injury in mice. These results also suggest that the EPF analysis is useful for the evaluation of plasticity in sensory functions in animal disease models.

## Findings

Primary afferent fibers have been classified into three major types, C-, Aδ-, and Aβ-fibers. To unveil the specific role of each of these fiber types in pain sensation, many *in vivo *models have been developed. In many cases, the animals' response to various painful stimuli, consisting of thermal, mechanical or chemical challenges, is measured as the output parameter, and each of these types of stimuli have proven to partly mimic clinical symptoms observed in patients and healthy subjects [1,2]. However, the mechanisms that lead from stimuli to response are unclear but is expected to consist of both direct and indirect actions [3,4], and a better understanding may lead to novel treatment strategies.

We recently developed a technique that measures the paw-flexion-response induced by extremely low doses of algogenics (algogenic-induced paw flexion or APF test) [5]. Combined with neonatal capsaicin-induced C-fiber elimination and spinal receptor antagonism, we categorized nociception into three types. Type 1 is induced by bradykinin, SP or histamine, while type 2 is induced by ATP. Furthermore, type 1 and 2 nociception are mediated through SP-NK1 and glutamate-NMDA receptor spinal transmission, respectively. However, they both are capsaicin-sensitive fibers [5]. This classification is similar to the schematic model proposed by Snider and McMahon [6]. In contrast to type 1 and 2 nociception, type 3 nociception induced by prostaglandin I_2_ receptor agonists is mediated by capsaicin-insensitive fibers and spinal transmission through glutamate-NMDA receptors, possibly reflecting Aδ-fiber signaling [5]. The physiological function of Aβ-fibers is thought to be conduction of innocuous tactile input, though they may possess nociceptive properties [7].

To evaluate the sensory transduction, especially concerning that of Aβ-fibers, we utilized the Neurometer^®^, an apparatus that selectively activates sensory neurons by use of sine-wave pulses of different frequencies. In humans, frequencies of 5, 250 and 2000 Hz activates C-, Aδ- and Aβ-fibers respectively. This test is widely used clinically to evaluate sensory function in patients suffering from peripheral neuropathic pain [8-11]. However, as the characterization of these stimuli-induced responses in animals remains to be clarified, we examined the flexor responses induced by the three distinct stimuli (5, 250 and 2000 Hz) in the EPF test, which is derived from the APF test [5,12]. Next, we also determined the functional responses in each nociceptor type in a murine nerve-injury model.

Male ddY mice weighing 20–22 g, were adapted to laboratory condition; 22 ± 2°C, 55 ± 5 % relative humidity and a 12-hour light/dark cycle with food and water ad libitum. All procedures were approved by Nagasaki University Animal Care Committee and complied with the recommendations of IASP [13]. The following drugs were used: capsaicin (Nacalai Tesque, Kyoto, Japan), MK-801 (Research Biochemical, USA) and CNQX (Sigma, USA). CP-99994 and capsaicin cream were generously provided by Pfizer Pharmaceuticals (Sandwich, Kent, UK) and Maruishi Pharmaceutical Co. (Osaka, Japan), respectively. All drugs except capsaicin were dissolved in physiological saline. Neonatal capsaicin or capsaicin cream treatments were carried out as previously reported [14-16]. Partial sciatic nerve ligation was performed under pentobarbital (50 mg/kg, i.p.) anesthesia, as described previously [16,17]. Tests were carried out 7 days after nerve ligation.

**Figure 1:**
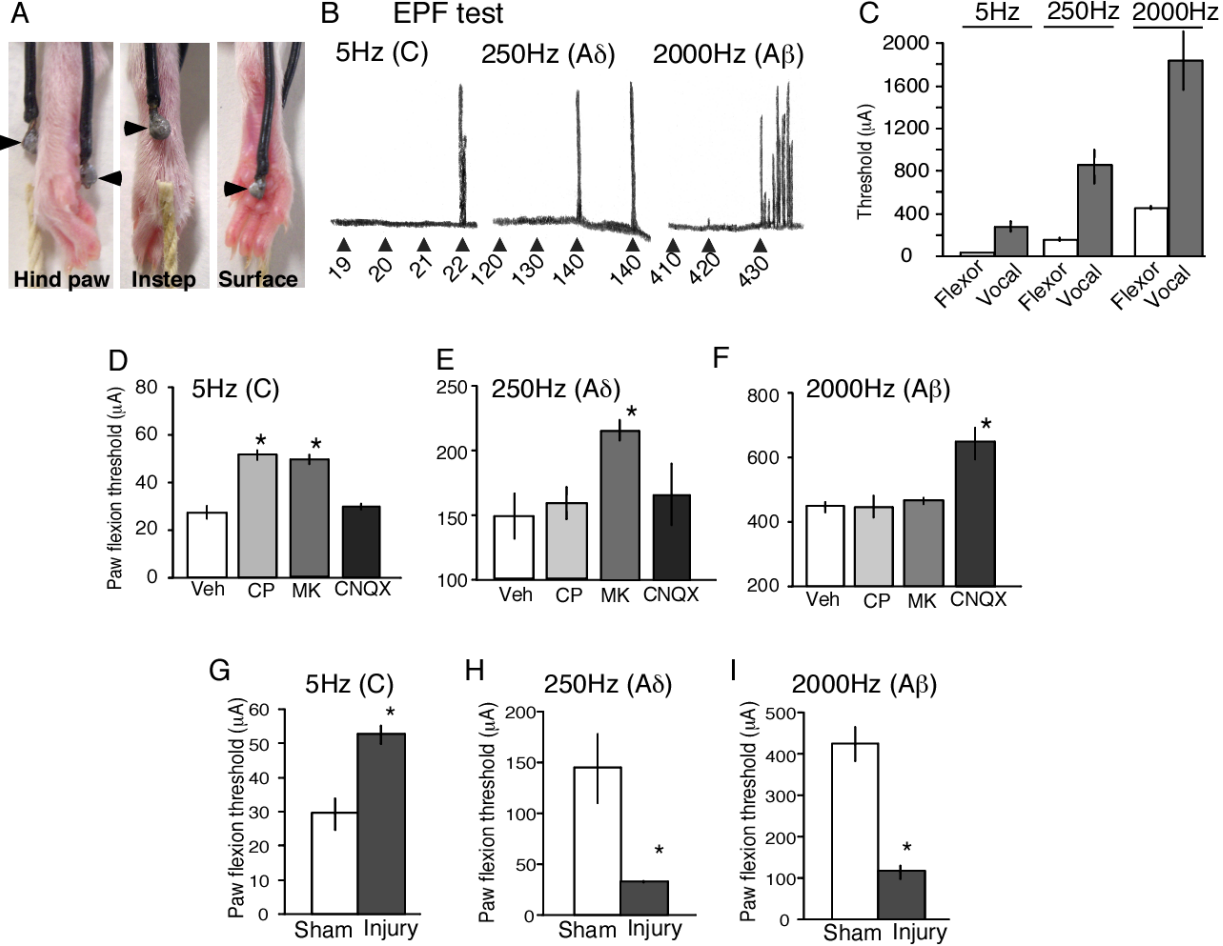
**Spinal transmission in flexor responses induced by three different sine-wave electrical stimuli. **(A) Attachment of electrodes to the right hind paw plantar surface and instep of the mice. Arrowheads indicate electrode. (B) Representative trace of each of three different sine-wave (5, 250 and 2000 Hz) stimulus-induced responses. The intensity of each stimulus was gradually increased. (C) Average electrical stimulation thresholds for vocalization and paw flexor responses. (D-F) Sensitivity of NK1, NMDA and AMPA/kainate antagonists on three different sine-wave (5, 250 and 2000 Hz) stimulus-induced responses in the EPF test. (G-I) Opposite effects on C- and A-fiber-mediated response by nerve injury in mice. Sensitivity was determined by EPF tests on day 7 after sciatic nerve injury. The data were analyzed using Student's *t*-test. Significance was set to *p < 0.05. Data in these figures are presented as means S.E.M. from experiments using at least 6 mice.

While tested, mice were suspended from a metal bar in a cloth sling, with their 4 limbs hanging free through holes. All limbs were tied with strings; 3 were fixed to the bench, while the 4th was connected to an isotonic transducer and recorder. Electrodes were attached to the right hind paw plantar surface and instep (Fig. 1A) and fastened with tape. Twenty minutes after attaching the electrodes the mice were calm and the chart was steady. Then, nerve stimuli with each of the three pulses (5, 250 and 2000 Hz) were applied using the "manual mode" of the Neurometer (Neurotron Inc., Baltimore, MD). The basal level of the chart line was the same from beginning to end in experiments. In this experiment, stimuli were applied with 5 min intervals. The minimum intensity (in μA) to induce a flexor response (EPF) or vocalization [18] was defined as the stimulus threshold.

Fig. 1B demonstrates the representative traces of flexor responses by 5, 250 and 2000 Hz stimulations. The averages thresholds to induce flexor responses by such stimulations were 26.0, 147.5 and 440.7 μA, respectively (Table 1). Repeated stimulations by 250 Hz induced consistent flexor responses (Fig. 1B), and so did the stimulation by 5 or 2000 Hz (data not shown). The thresholds for vocalization responses to 5, 250 and 2000 Hz stimulation were much higher than those for the flexor response (Fig. 1C).

**Table 1:** Capsaicin sensitivity. Neonatal capsaicin treatment and capsaicin cream reduced sensitivity measured as flexor responses to electrical stimulations with three different frequencies. *:*p *< 0.05. Data (μA) are presented as means ± S.E.M. from experiments using at least 6 mice.

	Veh-mice	Cap-mice	Basement	Cap cream
5 Hz	26.0 ± 2.6	95.8 ± 22.7 *	29.3 ± 4.6	74.5 ± 2.3 *
250 Hz	147.5 ± 17.8	155.4 ± 33.0	143.9 ± 34.4	158.0 ± 15.3
2000 Hz	440.7 ± 16.1	414.0 ± 18.9	423.7 ± 41.9	476.0 ± 43.7

Table 1 demonstrates the capsaicin-sensitivity. Only the threshold for 5 Hz stimulations was significantly increased by neonatal-capsaicin and capsaicin cream treatments, which degenerate and desensitize C-fibers, respectively [14,15], while those for 250 Hz and 2000 Hz were not. The characterization of spinal pain transmission is shown in Fig. 1D–1F. The treatment 20 min prior to EPF tests with CP-99994 or MK-801 (3 nmol, i.t.), an NK1 and NMDA receptor antagonist, respectively, significantly increased the threshold of the 5 Hz response while no effect was observed with CNQX (3 nmol, i.t.), an AMPA/kainate receptor antagonist (Fig. 1D). However, the 250 Hz threshold was increased by MK-801 but not by CP-99994 or CNQX pretreatment (Fig. 1E). Finally we found that the 2000 Hz threshold was increased by CNQX but not by CP-99994 or MK-801 pretreatment (Fig. 1F).

Next, we evaluated the functional responses of each of the primary sensory fibers in the partial sciatic nerve ligation model of neuropathic pain. The 5 Hz threshold was significantly increased on day seven after operation (Fig. 1G), while the 250 Hz and 2000 Hz thresholds were significantly decreased (Fig. 1H and 1I).

The present study demonstrates that the 5 Hz or C-fiber responses are mediated through capsaicin-sensitive fibers and SP-NK1 receptor as well as glutamate-NMDA receptor spinal transmission. These findings are in a good accordance with our previous studies using the APF test, in which C-fiber-mediated nociceptive responses are sensitive to the NK1 antagonist (CP-99994) or the NMDA receptor antagonist (MK-801) [5]. The 250 Hz-responses are mediated through capsaicin-insensitive fibers and glutamate-NMDA-receptor spinal transmission. As type 3 nociception is mediated by capsaicin-insensitive fibers and glutamate-NMDA-receptor spinal transmission [5], the 250 Hz stimuli may activate at least type 3 fibers. Furthermore, we found that the 2000 Hz response is mediated through capsaicin-insensitive fibers as well, but depends on glutamate-AMPA/kainate-receptor transmission.

It seems to be unique that MK-801, but not CNQX inhibited the nociceptive responses by Aδ (250 Hz) stimulation, since NMDA receptor activation is known to require depolarization through AMPA receptors for removing the Mg^2+ ^block. Regarding the lack of contribution of AMPA receptors to the NMDA receptor-mediated nociceptions, there are some other possibilities for selective NMDA receptor activation. In the presence of ephrinB2-EphB2 activation, glutamate-induced excitatory postsynaptic potential (EPSP) was blocked by MK-801, but not by CNQX [19]. In fura-2 imaging experiments, it was revealed that the [Ca^2+^]_i _increase in spinal lamina I neurons by electrical stimulations was blocked by D-AP5, an NMDA receptor antagonist, but not by CNQX [20]. From the fact that the single-channel conductance and open channel probability of NMDA receptor-mediated currents (~30 pS, ~10 msec) are longer than AMPA/kainate receptor-mediated one (2–10 pS, 2–5 msec), the NMDA receptor currents seem to be more liable to be cumulated to generate action potentials than AMPA receptor ones under the condition of repetitive stimulation by Neurometer, as shown in the present study.

The present findings firstly demonstrate that the spinal pain transmission through Aδ- or Aβ-fibers is differentially mediated through glutamate-NMDA receptors or glutamate-AMPA receptors, respectively. Such a distinct characterization would be attributed to the fact that we used low-intensity stimulation of 2000 Hz, since higher intensity stimulations (>2,200 μA) activates Aδ-fibers as well as Aβ-fibers, as seen in the recent electrophysiological study using Neurometer [21].

We also demonstrate that opposite changes in the sensitivity of C-fiber- and A-fiber-mediated responses were induced in mice after pretreated with partial sciatic nerve injury. These findings are consistent with our previous studies using APF test, in which type 1 nociceptive responses through C-fibers were lost, while type 3 nociceptive responses (possibly through Aδ-fibers) were enhanced by nerve injury [5,16]. The present findings provide further the evidence that nociceptive responses through Aβ-fibers are also enhanced. Although the underlying mechanisms remain to be clarified, reduced C-fiber responses are likely related to the decrease in dorsal horn SP-immunoreactivity in neuropathic pain models [17], while enhanced A-fiber responses are related to the up-regulation of calcium and sodium channels as well as to demyelination [22,23]. In conclusion, this is the first report of a murine *in vivo *characterization of the distinct responses by C-, Aδ- and Aβ-fiber stimuli using Neurometer. We found opposite effect on C- and A-fiber-mediated responses following partial sciatic nerve injury, suggesting that targeting A-fibers, responsible for neuropathic pain but not C-fibers, is a potential clinical treatment strategy.

## Abbreviations

SP: substance P, NK1: neurokinin 1, NMDA: N-methyl-D-aspartate, AMPA: α-amino-3-hydroxy-5-methyl-isoxozole propionic acid, MK-801: (+)-5-methyl-10,11-dihydro-5H-dibenzo [1, d,] cyclehepten-5,10-imine hydrogen maleate, CNQX: 6-cyano-7-nitroquinoxaline-2,3-dione, EPF: electrical stimulation-induced paw flexion
